# Constructing a Hierarchical Framework for Assessing the Application of Big Data Technology in Entrepreneurship Education

**DOI:** 10.3389/fpsyg.2020.551389

**Published:** 2020-09-11

**Authors:** Hongjia Ma, Chunting Lang, Yang Liu, Yang Gao

**Affiliations:** ^1^School of Management, Jilin University, Changchun, China; ^2^School of Economics and Management, Dalian University of Technology, Dalian, China

**Keywords:** entrepreneurship education, big data, sustainable development, hierarchical framework, new venture

## Abstract

The emergence of big data technology continues to innovate and change the world, bringing opportunities and challenges to all walks of life. Against the background of this era, traditional entrepreneurship education requires reform and innovation. This research attempts to explore the ways and practices of applying big data technology to entrepreneurship education so as to improve and perfect traditional entrepreneurship education and achieve its sustainable development. Based on classic theories, such as entrepreneurial theory, strategic management theory, and leadership theory, this paper develops a relatively systematic attribute system of entrepreneurship education under big data technology, comprehensively uses Fuzzy-DEMATEL and ISM methods to explore the relationship between different attributes and their importance, and finally constructs a hierarchical framework for the application of big data technology in entrepreneurship education. The results show that the attributes of entrepreneurship education under big data technology can be divided into four levels, each with different priorities and degrees of importance, and there are complex interactions and constraints among them. This study provides important guidance and suggestions for the development of entrepreneurship education and multiattribute decision-making management under the given resources, which is conducive to the sustainable development of entrepreneurs and new ventures.

## Introduction

The continuous innovation of modern technology and communication systems has ushered in the era of big data, which has brought new opportunities and challenges (Wang, 2018). Naturally, big data technology poses challenges to entrepreneurs, and traditional entrepreneurship education needs transformation and innovation. Yang et al. (2014) points out that entrepreneurship education has important strategic significance and educational value. Also, an effective evaluation system is vital to guide, shape, and assess education. Further, Zheng (2019) points out that big data mining can play a very important role in establishing an entrepreneurship education system. Hence, research on entrepreneurship education with the help of big data technology is of great significance and requires a systematic evaluation framework.

Although previous studies on entrepreneurship education have been valuable, they also have limitations. First, previous studies focus more on entrepreneurship education in colleges and universities (Li, 2017; Chen Y. et al., 2018; Gianiodis and Meek, 2019). However, some facets of education in colleges and universities might not apply to entrepreneurship education in social institutions. Second, most scholars only explore traditional entrepreneurship education, and research on entrepreneurship education combined with big data technology is rare. For example, García-Rodríguez et al. (2017) explores the impact of the business model on the entrepreneurial potential of college students in entrepreneurship education. Although Zheng (2019) introduces big data into entrepreneurship education, the study focuses on logistics management and lacks applications in other functions. Furthermore, it is particularly important to emphasize that most studies on entrepreneurship education analyze issues qualitatively and propose corresponding countermeasures (Hao, 2017; Hua, 2019), and empirical research is relatively rare, which is not conducive to the long-term development of entrepreneurship education. Finally, when new technologies, such as big data, are embedded, entrepreneurship education research in the new context lacks a systematic framework. Shah et al. (2019) points out that a hierarchy contributes to improving corporate performance and plays a key role in implementing strategies. In addition, hierarchical jumps can improve management efficiency and further promote sustainable development (Sarfraz et al., 2019). Therefore, it is of great significance to explore a systematic hierarchical framework for entrepreneurship education. Because the different levels of entrepreneurship education are not isolated and are collaborative and mutually influential, the research also needs to reveal the potential influence and development paths between different levels. This work can provide a vital foundation for subsequent research on entrepreneurship education.

Based on the above, this paper carried out the following research. First, to construct a systematic attribute system of entrepreneurship education, the research focused on theories of entrepreneurship, strategic management, and leadership, extracting a total of 12 aspects, including business opportunities, monetary decisions, financial management, and legitimacy (institution, business ethics, and corporate culture) that are emphasized in entrepreneurship theory; value chain management (production management, logistics management, marketing management, and human resource management) emphasized by strategic management theory; and leadership traits and behavior (psychology, leadership) emphasized by the theories of leadership. After that, considering the application of big data technology in entrepreneurship education, 29 criteria were proposed from the 12 aspects above. Finally, we explored the development paths of entrepreneurship education under big data technology, and a hierarchical framework was built, which takes entrepreneurs as the core and focuses on sustainable development of entrepreneurs and new ventures and is not limited to entrepreneurship education in colleges or social institutions. To make complex decisions among multiple attributes, the mutual constraints and influences between attributes should be considered in this part. This study used fuzzy set theory and the decision-making trial and evaluation laboratory (DEMATEL) to identify causality interrelationships and explore the degree of importance among attributes. After that, the interpretative structural modeling (ISM) method was used to divide the levels, reveal development paths, and finally construct a hierarchical framework.

The results of this study indicate that the attributes of entrepreneurship education under big data can be divided into four levels with mutual constraints and influences between each level. Among them, business opportunities, institutions, and psychology are located on the first level of the ISM model, and these are primary and decisive aspects of entrepreneurship education. Leadership and financial management constitute the second level, stressing a focus on the leadership behavior of entrepreneurs and the assessment and improvement of a financial situation. The third level centers on the value chain, which is the development stage of formal entrepreneurial activities and has an important connection with the value-creating aspect of the enterprise. The last level includes business ethics, corporate culture, and human resource management. The realization of this level needs to undergo a complicated process, and it is affected by aspects of the first three levels.

This research complements existing studies of entrepreneurship education. Systematic and forward-looking, the hierarchical theoretical framework reflects not only the multifaceted and complex nature of the entrepreneurial process but also the embeddedness of new technologies. The results of this paper provide important guiding suggestions for entrepreneurship education, which are conducive to the transformation, innovation, and sustainable development of entrepreneurship education.

## Literature Review

### Big Data Technology

Due to the rapid development of communication and information technology, research has entered the era of big data. With the improvement in information society and enterprises, the influence of big data technology is expanding, which makes people begin to realize the huge economic benefits and social value of big data. Gartner, a big data research institution, believes that “big data” needs new processing modes to have stronger decision-making power, insight, and discovery ability as well as process optimization ability to adapt to the massive, high growth rate and diversified information assets. Specifically, big data covers a series of data generated by Internet behavior, including the preferences and intentions of producers and users as well as data related to non-traditional structures. It is characterized by great variety, huge amounts of information, and extremely fast production and update speeds. The strategic significance of big data technology lies not only in mastering huge amounts of information, but also, more importantly, the acquisition, integration, and specialized processing and analysis technology of large-scale data (Wang and Hajli, 2017). Using the new processing modes, big data technology can achieve stronger decision-making power and insight ability and create huge economic benefits and social value through the processing of massive and diversified information assets.

Nowadays, big data technology is applied in many fields, such as e-commerce, finance, manufacturing, transportation, social security, smart medical treatment, education, and so on, providing services and assistance in people’s daily lives and the operation and management of enterprises. For enterprises, big data is a strategic asset, which can provide massive information for the operation of enterprises, improve efficiency, save costs, and enhance competitiveness and strategic decision-making ability (Ghasemaghaei et al., 2018; Rijmenam et al., 2019). At the same time, big data technology plays an important role in the innovation and promotion of education. Klašnja-Milićević et al. (2017) point out that the application of data science and big data analysis in the field of education is of great significance and believe that a high-quality and multifunctional educational platform could be constructed through big data technology. This research shows that the application of big data technology in the field of entrepreneurship education, on the one hand, can improve the existing education mode, grasp the focus of entrepreneurship education, and carry out entrepreneurship education work in a more targeted manner; on the other hand, learning and mastering big data technology provides guidance and help for the management and development of new ventures.

### Big Data Technology and Sustainable Development of Entrepreneurship Education

Entrepreneurship education is a type of education that cultivates people’s entrepreneurial consciousness, thinking, skills, and other qualities, ultimately enabling the educated to have a certain entrepreneurial ability. Entrepreneurship education is of great significance to the development of entrepreneurs and enterprises, and it is essential for meeting economic and social goals (Wu et al., 2018). With the arrival of the era of big data, traditional entrepreneurship education faces major challenges and needs development and innovation urgently.

In 1987, the World Commission on Environment and Development defined sustainable development as “development that meets the needs of the present generation without jeopardizing the ability of future generations to meet their needs.” Sustainable development is a new concept of development, whose main body can be economy, resources, environment, science and technology, education, and many other aspects. Among them, the sustainable development of education is an important component of the ability of sustainable development and plays a significant role in achieving the sustainable development of society (Findler et al., 2019). A sustainable education system attaches importance to educational reform, effective use of educational resources, and the strengthening of moral standards for sustainable development. This kind of education not only includes the form of school education, but also covers a wide range of subtle social education.

The sustainable development of entrepreneurship education is mainly composed of the sustainable development of entrepreneurs and of new ventures. On the one hand, the sustainable development of entrepreneurs emphasizes taking entrepreneurs as the core and improving entrepreneurial skills and the comprehensive quality required by entrepreneurs. The use of big data technology to collect and analyze massive cases and information will help to achieve the sustainable development of entrepreneurs. On the other hand, the sustainable development of new ventures means the survival, management, and long-term development of new ventures, which is also crucial for entrepreneurship and can be achieved by educating entrepreneurs to use big data technology reasonably and effectively to improve enterprise management. It can be seen that integrating big data technology into traditional entrepreneurship education can optimize and improve entrepreneurship education and promote the sustainable development of entrepreneurs and new ventures.

### Proposed Attributes

This study has developed 12 aspects and 29 criteria related to entrepreneurship education with the use of big data technology. The specific criteria and explanations are shown in Table 1.

**TABLE 1:** Proposed attributes.

Aspects	Criteria	Explanation
A1 Production management	Economies of scale (C1)	Calculate the optimal scale of production to reduce production costs.
	Learning effect (C2)	Monitor and trace production processes to establish a more comprehensive learning system and move the experience curve down.
	Quality control (C3)	Trace the source of product defects to reduce product quality issues.
A2 Logistics management	Warehouse management (C4)	Realize collaborative management of inventory through the information interaction between the warehousing and sales departments.
	Transport management (C5)	Find the optimal transportation solution to reduce transportation costs.
A3 Marketing management	Distribution channel (C6)	Monitor the status of sales channels (sales volume, costs, human resources, etc.) and establish a sound distribution plan.
	Customer relationship management (C7)	Collect customer after-sales data to provide feedback and grasp the causes of problems to carry out targeted customer management.
A4 Human resource management	Recruitment management (C8)	Predict the supply and demand for human resources reasonably and match the most suitable talents for positions.
	Training and development (C9)	Provide targeted training based on the future development of employees by analyzing data.
	Performance management (C10)	Build a comprehensive performance evaluation model and provide diverse reference data.
A5 Psychology	Failure tolerance (C11)	Count the failures of entrepreneurship and analyze failure causes and countermeasures.
	Risk-taking (C12)	Collect information on entrepreneurial failure rates and guide entrepreneurs to increase their awareness of risk-taking.
	Self-efficacy (C13)	Analyze and identify factors affecting the self-confidence of entrepreneurs to cultivate the self-efficacy.
A6 Monetary decisions	Investment (C14)	Master different investment channels, assess risks and feasibility of investment projects, and optimize investment plans.
	Fundraising (C15)	Grasp different channels, risk links, and risk types of fundraising.
A7 Business opportunities	Identifying opportunities (C16)	Collect and analyze data to seize the opportunities to arbitrage or optimize products and services.
	Discovery opportunities (C17)	Use big data technology to understand and discover opportunities for which either their supply side or demand side is missing.
	Creative opportunities (C18)	Collect data to grasp the development trends of socioeconomy to seize creative opportunities.
A8 Business ethics	Explicit ethics (C19)	Understand stakeholders’ concerns about business ethics and issue reports to make business ethics visible.
	Implicit ethics (C20)	Collect and analyze corporate social responsibility cases to cultivate employees’ ethical responsibility.
A9 Corporate culture	Corporate culture building (C21)	Grasp the inheritance of culture and create a unique corporate culture by collecting and studying cases of successful corporate culture.
	Corporate culture feedback (C22)	Collect public feedback on corporate culture to develop a healthy corporate culture.
A10 Institution	Formal institutional entrepreneurship (C23)	Find the needs of formal systems and grasp the types of formal institutional entrepreneurship.
	Informal institutional entrepreneurship (C24)	Find institutional defects and grasp various forms of informal institutional entrepreneurship.
A11 Financial management	Financial forecast (C25)	Predict future financial status of the company to make smart financial decisions and improve the financial situation.
	Financial risk (C26)	Screen and analyze data to find harmful factors to avoid or address financial risks reasonably.
	Business integration (C27)	Strengthen data interaction between the finance department and other business departments to achieve business integration.
A12 Leadership	Transactional leadership (C28)	Record and analyze employee feedback on transactional leadership behaviors to find the best way for transactional leadership.
	Transformational leadership (C29)	Collect and study successful transformational leadership cases to better coach employees.

#### Production Management

Production is an important part of the daily operation of enterprises, and it is also one of the primary activities on the value chain. Reducing production costs and ensuring product quality are difficult in traditional production processes. The use of big data technology can enable enterprises to achieve economies of scale (C1) in production. Entrepreneurs can use big data technology to calculate the optimal scale of enterprise production to reduce production costs. In addition, using big data technology can monitor production links, trace production processes, and establish a more comprehensive learning system to shift the experience curve down and achieve the learning effect (C2). Finally, using big data technology to find the source of product defects can ensure that the product is under quality control (C3) while in the production processes, thereby reducing product quality problems and optimizing the production processes of the enterprise.

#### Logistics Management

In traditional logistics management, companies lack a comprehensive understanding of logistics costs, which may lead to misallocation of resources and a large amount of capital consumption (Yan, 2019). Big data technology provides more information support for enterprise logistics cost management. Applying information technology to enterprise logistics cost management is conducive to realizing the information management and structural optimization of the logistics function (Li and Zhao, 2019).

Through the establishment of a real-time feedback system between the warehousing and sales departments and the interaction of data and information, a network of upstream and downstream links can be formed to achieve collaborative management of inventory (C4). Apart from this, entrepreneurs can build a logistics information platform, extract more valuable information from massive data, and find the optimal transportation solution (C5) to reduce transportation costs (Li and Zhao, 2019).

#### Marketing Management

As a daily operational management capability, marketing is part of the entrepreneurial capabilities that entrepreneurs must cultivate (Yang et al., 2014). Nowadays, traditional marketing activities of enterprises struggle to grasp the increasingly complex customer demand and customer psychology, creating an urgent need for change. Big data technology can help companies build an innovative road for marketing management and improve marketing effectiveness (Han, 2019).

Channel selection has become an important part of consumers’ complex decision-making processes (Han, 2019). Using big data technology to monitor the status of distribution channels (sales volume, costs, human resources, etc.) can establish a reasonable and sound sales plan (C6). Through the big data integration system, enterprises can obtain business-related data sources to understand customers’ behavior and provide feedback, which will help improve products and services and get them closer to the target market in order to meet the needs of consumers and enable more effective customer relationship management (C7) (Kubina et al., 2015; Anshari et al., 2019).

#### Human Resource Management

With the rapid development of the Internet and associated technologies, traditional human resource management strategies struggle to satisfy enterprises’ practical needs (Shen, 2015). Relying on the convenience brought by big data, enterprises can speed up the innovation of human resource management and change management thinking to achieve better development (Zhang, 2019).

Traditional recruitment has low effectiveness. With the help of big data technology, information on employees and jobs can be integrated, and a “personnel database” can be established to predict the supply and demand for human resources. Through processing and analyzing the data, the most suitable talents can be found to improve the degree of matching for positions and employees, which can achieve effective recruitment (C8) (Zhang and Xu, 2018). Furthermore, the use of big data can measure the comprehensive ability of employees, predict their future development, and conduct targeted training (C9) to unlock their potential and improve their motivation. Finally, big data technology also provides diverse reference data and more effective evaluation models for performance evaluation (C10) (Li, 2018).

#### Psychology

The success of entrepreneurship depends on the characteristics and abilities of entrepreneurs. Therefore, entrepreneurship education needs to focus on cultivating the psychological qualities of entrepreneurs. However, the relevant content in this area is relatively defective in entrepreneurship education, and the entrepreneurship education course has not yet included scientific planning (Hao, 2017).

Big data technology can be used to deeply analyze the complex psychology of entrepreneurs and guide entrepreneurs to cultivate specific psychological qualities. Entrepreneurship is not a smooth process. Entrepreneurs must have the ability to endure setbacks (Hao, 2017). Using big data technology to analyze and study the causes and countermeasures of entrepreneurial failures can help to improve the failure tolerance of entrepreneurs (C11). In addition, entrepreneurs must develop a risk-taking capability to survive the competition (Cui et al., 2016). Therefore, increasing the risk-taking awareness of entrepreneurs (C12) is also part of psychological education, which can be achieved by collecting information on entrepreneurial failures and providing guidance to entrepreneurs. Finally, self-efficacy (C13) reflects the confidence that an individual has in their abilities. Successful entrepreneurs are usually convinced that they can bring any activity to a successful ending. Also, they feel that they can control their success, which does not depend on others (Ismail and Zain, 2015). Using big data technology to understand the self-confidence of entrepreneurs and analyze its influencing factors can help to cultivate the self-efficacy of entrepreneurs.

#### Monetary Decisions

With increasing economic globalization and development and innovation in information systems, the monetary decision-making environment of enterprises is changing rapidly. On the one hand, the continuous emergence of new business and products has made the decision making of enterprises more and more complicated. On the other hand, the application of big data technology has revolutionized the business model in the monetary field, challenging the traditional monetary model and gradually developing in the direction of Internet finance (Yu, 2015; Zhang et al., 2015). All of these have affected the monetary decisions of enterprises accordingly.

Ensuring scientific quality and predictability is difficult given the traditional monetary decision making of enterprises. With big data technology, companies could discover value from the data and apply it directly to major business decisions, making monetary decisions more scientific. By using big data technology, entrepreneurs can see channels of investment (C14) and fundraising (C15) more comprehensively, and through more accurate assessments of risks and feasibility, smart investment and fundraising can be realized to optimize monetary decisions (Xia and Zhou, 2015).

#### Business Opportunities

Research on opportunity identification and development occupies an important position in the field of entrepreneurship research. Entrepreneurship is a process of chasing and realizing business opportunities. The real entrepreneurial process begins with the discovery of opportunities by entrepreneurs (Shane and Venkataraman, 2000). The identification, acquisition, and integration of entrepreneurial opportunities are the prerequisites and necessary conditions for starting a new business. Furthermore, the ability to identify opportunities is an important dimension of entrepreneurial abilities. Obtaining a strong opportunity recognition ability gives the enterprise a first-mover development to create opportunities for the realization of corporate goals (Zhou and Gao, 2019).

Relying on big data technology, the risks and types of business opportunities can be evaluated systematically. Here are three types of opportunities that can be developed by entrepreneurs: identifying opportunities, discovery opportunities, and creative opportunities. The development of identifying opportunities (C16) can be achieved by collecting huge amounts of data to find opportunities for arbitrage or optimizing and improving products and services to better meet people’s needs. Discovery opportunities (C17) emphasize situations in which either supply or demand is missing. Massive cases could help entrepreneurs grasp the existence and differences of supply and demand-oriented opportunities. Finally, relevant data and information on changes in the social environment could predict the development trends of socioeconomy and related industries effectively, which will help to capture creative opportunities (C18).

#### Business Ethics

In today’s society, corporate social responsibility (CSR) has seeped into every aspect of the enterprise. CSR has not only changed the social participation and social life of the enterprise but also the relationship between enterprises and their stakeholders. The success of an enterprise depends on the help and support of a series of stakeholders. To achieve sustainable development, enterprises must be economically responsible to shareholders and socially responsible to other stakeholders (such as customers, employees, community members, etc.), which is a reflection of their business ethics. Therefore, it is necessary to educate entrepreneurs to make them aware of the importance of CSR and business ethics. Traditionally, it is difficult for companies to achieve real ethical externalization and internalization. Big data technology makes the explicit and implicit ethics of companies visible and helps them to better realize those ethics. Externally, entrepreneurs can use big data technology to grasp the concerns of different stakeholders on business ethics and issue targeted reports to make corporate ethics visible (C19) (Li et al., 2018). Internally, collecting and studying cases related to corporate social responsibility could develop employees’ sense of ethical responsibility and internalize corporate ethics (C20).

#### Corporate Culture

Corporate culture is the soul of corporate survival and development and a powerful driving force for sustainable development of enterprises (Shen, 2018). In the context of economic globalization and rapid development of science and technology, optimizing and upgrading corporate culture constantly, creating the innovative corporate culture with core values, ensuring long-term development, and enhancing core competencies of enterprises are necessary for the survival and development of enterprises in domestic and international markets (Chen, 2017). Also, enterprises need to seize the opportunities brought by big data and accelerate the improvement and adjustment of the aspects of corporate culture (Chen G. et al., 2018).

Using big data technology to collect cases of corporate culture can enable entrepreneurs to learn outstanding cultures of different industries and fields and to grasp the inheritance of culture, thereby creating a unique corporate culture (C21). In addition, traditional corporate culture-building lacks close connection with the public, and big data technology can help entrepreneurs collect public attitudes and evaluations of corporate culture and establish a cultural feedback mechanism (C22), which can help improve and develop corporate culture.

#### Institution

There is a close relationship between the institution and entrepreneurship. A reasonable institution can encourage entrepreneurs to devote themselves to entrepreneurial activities and promote the increase of social wealth and economic development. Ahlstrom and Bruton (2002) indicate that entrepreneurial enterprises could not only adapt to existing institutional environments, but also create a relatively favorable institutional environment for themselves by changing certain conditions. Institutional entrepreneurship is a new topic in strategic management in recent years, and it refers to entrepreneurial behavior processes in which entrepreneurs mobilize resources to change existing institutions or create new institutions under the institutional framework; establish and promote the rules, values, beliefs, and behavior models that organizations need to gain recognition; and create, develop, and utilize profitable opportunities (Maguire et al., 2004; Greenwood and Suddaby, 2006). Institutional entrepreneurship is not a one-time act, but a development process that is dynamic and facilitates the complete transformation of the institutional framework. Given the dynamics and complexity of institutional entrepreneurship, we can use big data technology to help entrepreneurs grasp the characteristics and forms of institutional entrepreneurship conveniently. Using big data technology to find the demand for formal systems helps entrepreneurs grasp the types of formal institutional entrepreneurship (C23) and start a business that meets the demand of the systems. Furthermore, big data technology can help entrepreneurs understand institutional defects and various forms of informal institutional entrepreneurship (C24), which provides them with more entrepreneurial options.

#### Financial Management

Big data technology has brought opportunities for the innovation and development of financial management in order to further develop enterprises. Introducing big data technology into financial management can greatly improve the efficiency of financial management and gradually enhance the competitive advantage of enterprises (Wang, 2019). Using big data technology to predict future financial status, operating results, and cash flow prospects of the company (C25) can help entrepreneurs make financial decisions that are more conducive to the development of the company and improve the financial situation in a targeted manner. By screening and analyzing data to find harmful factors, entrepreneurs can avoid or address financial risks rationally (C26). In addition, big data can promote information interaction between different departments, which helps to integrate the financial department with other business departments, such as production, sales, human resources, etc. (C27) (Cheng and Dong, 2015).

#### Leadership

Among key factors affecting corporate operation and development, leadership is considered to be critical. The leadership behavior of entrepreneurs plays a decisive role in entrepreneurial and innovative capabilities. Zhu et al. (2005) point out that scientific leadership could promote the organizational construction and cultural atmosphere, thus exploring the creativity of the organization and improving the innovation efficiency. Hassan et al. (2018) believe that leadership effectiveness is crucial to the development of educational institutions and is mainly influenced by leadership practice and style.

Longshore and Bass (1987) first put forward the theory of transactional and transformational leadership. They divided leadership into two dimensions: transactional and transformational leadership. Transactional leadership behavior (C28) refers to the interaction between leaders and subordinates through a large number of exchanges and implicit contracts. Leaders guide subordinates mainly through rewards and fulfill promised rewards after they complete their tasks. The whole relationship process is like a transaction. Using big data technology to record and analyze employees’ feedback on transactional leadership behaviors will help entrepreneurs find the best way and improve their transactional leadership.

Transformational leadership behavior (C29) adopts visionary and creative leadership behavior and focuses on the establishment of emotional ties with followers, thus creating higher value (García-Morales et al., 2012). Transformational leaders convey their ideas and values to subordinates, transmit organizational goals and common missions to subordinates, and motivate them to stimulate inspiration and potential so that they can make the greatest efforts to achieve the organizational goals. Collecting and studying successful transformational leadership cases helps entrepreneurs to guide and support employees so that they can make their best efforts to achieve the overall goals of the organization.

Starting from classic management theories, such as entrepreneurship, leadership, and strategic management theory and combing the existing literature, the research has sorted out the attribute system of big data entrepreneurship education, which includes 12 aspects and 29 criteria. Next, fuzzy-DEMATEL and ISM methods are used to evaluate the complex relationship between attributes and construct a hierarchical framework.

## Materials and Methods

### Selection of Research Methods

The purpose of this study is to explore how to apply big data technology in the field of entrepreneurship education to improve and perfect traditional entrepreneurship education. Based on the theories of leadership, entrepreneurship, and strategic management and a review of the existing literature, 12 aspects and 29 criteria for entrepreneurship education under big data technology have been developed as the attribute system, and the specific practices of applying big data technology to entrepreneurship education have been systematically expounded, which is of guiding significance for the development of entrepreneurship education. However, the study also needs to further clarify the complex interrelations and importance of various aspects and criteria so as to ensure that more reasonable and effective multiattribute decision making can be carried out under the condition of limited resources, and the key aspects of entrepreneurship education can be better grasped. A fuzzy-DEMATEL hybrid method is adopted to analyze and evaluate attributes, which can clearly reveal the causal relationship between attributes and their importance through the values of centrality, causality, and the cause-and-effect diagram and clarify the important driving aspects of entrepreneurship education. In addition, the research needs to construct a more explicit hierarchical framework to simplify and clarify the complex interrelations among the attributes of entrepreneurship education to facilitate the overall control and rational decision making of entrepreneurship education by managers.

### Fuzzy-DEMATEL

Fuzzy mathematics based on fuzzy set theory is used to analyze the fuzzy degree of feature relevance. It is a method of simulating the human brain to process fuzzy information. The triangular fuzzy number (TFN) provides an effective means to quantify human linguistic preferences into computable form (Opricovic and Tzeng, 2004). Fuzzy set theory transforms qualitative language into quantitative data and overcomes the problem of expert linguistic preferences (the uncertainty caused by expert judgment), thus reducing the error and improving the credibility of the analysis results, which can provide a more valuable reference for managers’ decision making (Du et al., 2020).

The DEMATEL method is a decision-making tool based on graph theory and matrix calculation. It is used to analyze the importance of system factors and help to plan and solve problems (Lin and Tzeng, 2009). DEMATEL can be used to explore the relationship and influence degree of various factors affecting the evaluation object and reveal the causal relationship and importance of attributes so as to better evaluate problems and management decisions (Büyüközkan and Çifçi, 2012).

In this paper, a fuzzy-DEMATEL hybrid method is adopted to analyze and evaluate the relationship and importance between 12 aspects and 29 criteria for entrepreneurship education under big data technology, which not only solves the uncertainty of expert linguistic preferences, but also retains the practical and effective advantages of traditional DEMATEL method in factor identification. Specific steps are as follows:

Step 1: For the problem under study, build a system of factors, set to F1, F2,…, Fn.

Step 2: Determine the influence relationship between two factors by the expert scoring method and express it in matrix form. Invite experts to use the language operators “no influence (N),” “very low influence (VL),” “low influence (L),” “high influence (H),” and “very high influence (VH)” to evaluate the relationship between factors. Through the semantic transformation table shown in Table 2, the original expert evaluation is converted into TFNs $w_{ij}^{k}=\left(a_{1ij}^{k},a_{2ij}^{k},a_{3ij}^{k}\right)$ to represent the fuzzy weight of the *i*th factor that affects the *j*th factor evaluated by the *k*th expert.

**TABLE 2:** Semantic transformation table.

Linguistic variables	TFN
N (No influence)	(0, 0, 0. 2)
VL (Very low influence)	(0, 0. 2, 0. 4)
L (Low influence)	(0. 2, 0. 4, 0. 6)
H (High influence)	(0. 4, 0. 6, 0. 8)
VH (Very high influence)	(0. 8, 1, 1)

Step 3: Converting the fuzzy data into crisp scores (CFCS) method is used to defuzzify the initial value of the expert score and obtain the direct relation matrix Z that reflects the direct effect between the factors, including the following four steps:

(1) Normalization:

(1) $$xa_{1ij}^{k}=\left(a_{1ij}^{k}-\min a_{1ij}^{k}\right)/\Delta_{\min}^{\max}$$

(2) $$xa_{2ij}^{k}=\left(a_{2ij}^{k}-\min a_{1ij}^{k}\right)/\Delta_{\min}^{\max}$$

(3) $$xa_{3ij}^{k}=\left(a_{3ij}^{k}-\min a_{1ij}^{k}\right)/\Delta_{\min}^{\max}$$

(2) Compute left-side (ls) and right-side (rs) normalized values:

(4) $$xls_{ij}^{k}=xa_{2ij}^{k}/\left(1+xa_{2ij}^{k}-xa_{1ij}^{k}\right)$$

(5) $$xrs_{ij}^{k}=xa_{3ij}^{k}/\left(1+xa_{3ij}^{k}-xa_{2ij}^{k}\right)$$

(3) Calculate the crisp values:

(6) $$x_{ij}^{k}=\left[xls_{ij}^{k}\left(1-xls_{ij}^{k}\right)+xrs_{ij}^{k}xrs_{ij}^{k}\right]/\left[1-xls_{ij}^{k}+xrs_{ij}^{k}\right]$$

(7) $$z_{ij}^{k}=\min a_{1ij}^{k}+x_{ij}^{k}\times \Delta_{\min}^{\max}$$

(4) Calculate the average crisp values:

(8) $$z_{ij}^{k}=\left(z_{ij}^{1}+z_{ij}^{2}+\cdots +z_{ij}^{k}\right)/n$$

Step 4: Normalize the direct relation matrix Z to obtain the normalized direct relation matrix G:

(9) $$\lambda =1/\max_{1\leq i\leq n}\sum_{j=1}^{n}z_{ij},G=\lambda Z$$

Step 5: According to Equation (10) below, the total relation matrix T is obtained. (E is the identity matrix):

(10) $$T=G{\left(E-G\right)}^{-1}$$

Step 6: Based on the total relation matrix, the driving power (D) and dependence power (R) can be obtained. The elements in the matrix T are added by rows to obtain driving power Di, which represents the comprehensive influence of the factor in this row on all other factors. The elements in the matrix T are added by columns to obtain dependence power Ri, which represents the comprehensive influence of all other factors on the factor in this column. The equations are as follows:

(11) $$Di=\sum_{j=1}^{n}t_{ij}\left(i=1,2,\ldots ,n\right)$$

(12) $$Ri=\sum_{i=1}^{n}t_{ij}\left(i=1,2,\ldots ,n\right)$$

(D+R) represents the magnitude of the effect of the factor, which is called centrality (m). (D−R) reflects the causal relationship between the factors, which is called causality (n). If the causality is positive, it means that the factor has more effect on other factors and belongs to the cause group; conversely, if it is negative, it means that the factor is more affected by other factors and belongs to the effect group. The equations are as follows:

(13) $$m_{i}=D_{i}+R_{i}\left(i=1,2,\cdots ,n\right)$$

(14) $$n_{i}=D_{i}-R_{i}\left(i=1,2,\cdots ,n\right)$$

### ISM

The ISM method is used to classify the system structure to transform the ambiguous ideas and views into an intuitive model with structural relationships (Beikkhakhian et al., 2015). The fuzzy-DEMATEL method is usually used to evaluate the complex interrelation between factors at the micro level, and the ISM method focuses more on the macro level, which can decompose the complex system into subsystems and reveal the relationship between attributes more clearly and intuitively. This paper uses the ISM method to divide the attribute system of entrepreneurship education under big data technology into 4 levels, illustrates the influence paths between different levels, and finally, constructs a hierarchical theoretical framework. In the above fuzzy-DEMATEL analysis process, the total relation matrix is obtained. However, the total relation matrix T only reflects the mutual influence relationship between different factors, and the influence of the factors on itself has not been taken into account. Therefore, it is necessary to calculate the overall relation matrix that reflects the overall influence relationship of system factors. The calculation equation is

(15) $$H=T+E=h_{\text{ij}}$$

Next, the threshold λ is introduced to eliminate redundant information and obtain the most simplified matrix. Based on the trial calculation, the threshold calculation models that best fits the research is obtained. The equation is:

(16) λ=α+β

where α and β are the mean and standard deviation of all elements in the total relation matrix T, respectively.

The threshold is used to remove the redundant factors, and the reachability matrix M is obtained. The equations are as follows:

(17) M=[mij,n*n(i=1,2….n;j=1,2….n)

(18) $$m_{ij}=\left\{ \begin{array}{c} 1,h_{i,j}\geq \lambda \\ 0,h_{i,j}<\lambda \end{array}\left(i=1,2\ldots ,n;j=1,2,\ldots ,n\right)\right.$$

In Equation (18), 1 means there is a direct effect between the two factors, and 0 means there is no direct effect between the two factors.

Next, the reachability set L(fi), antecedent set P(fi), and intersection set C(fi) are obtained by hierarchical processing. The equation is:

(19) $$\text{C}\left({\text{f}}_{\text{i}}\right)=L\left({\text{f}}_{\text{i}}\right)\cap \text{P}\left({\text{f}}_{\text{i}}\right)$$

Finally, the ISM model is determined by the reachability set and intersection set.

## Results

Based on a review and analysis of the literature, 12 aspects and 29 criteria are summarized in this paper. Seven experts were interviewed, and their opinions on the impact relationship between these aspects and criteria were obtained by scoring.

All the experts have been engaged in the field of entrepreneurship education for more than 10 years and have rich practical experience. We introduced the purpose of the study to the experts and explained the connotation of attributes. Seven experts discussed the proposed attributes over and over again to ensure the reliability of the study. Then, the experts evaluated the degree of influence between attributes and filled in the questionnaires. This process takes the form of individual face-to-face interviews to ensure that the experts’ judgments are not affected. Finally, the original data in the questionnaires are processed to get the research results.

According to the CFCS method, the original data of 12 aspects are processed to obtain the direct relation matrix of the aspects as shown in Table 3.

**TABLE 3:** The direct relation matrix of the aspects.

	A1	A2	A3	A4	A5	A6	A7	A8	A9	A10	A11	A12
A1	0.0000	0.3790	0.1567	0.5694	0.2361	0.2044	0.3472	0.1250	0.2044	0.3948	0.2202	0.1726
A2	0.2679	0.0000	0.4266	0.5694	0.0139	0.2679	0.3472	0.0456	0.0774	0.2679	0.3313	0.3472
A3	0.2361	0.2996	0.0000	0.1567	0.2361	0.1567	0.3790	0.5694	0.5377	0.1250	0.1567	0.2361
A4	0.1726	0.0139	0.1250	0.0000	0.3472	0.0139	0.1091	0.2361	0.5694	0.2361	0.0139	0.2361
A5	0.1409	0.1409	0.3313	0.3948	0.0000	0.3472	0.5377	0.5060	0.3472	0.3472	0.3472	0.5694
A6	0.2044	0.2361	0.2679	0.1567	0.1885	0.0000	0.1726	0.4425	0.5694	0.1409	0.2520	0.2520
A7	0.5377	0.3472	0.5377	0.2520	0.3472	0.3472	0.0000	0.3472	0.2520	0.3313	0.5694	0.5694
A8	0.0139	0.2361	0.2996	0.0933	0.2361	0.1567	0.2044	0.0000	0.1250	0.2361	0.1250	0.1250
A9	0.1567	0.0139	0.0774	0.2520	0.2520	0.0456	0.0456	0.2044	0.0000	0.0139	0.1250	0.1250
A10	0.2361	0.2361	0.3472	0.3313	0.2361	0.5694	0.5694	0.2361	0.3472	0.0000	0.5694	0.5218
A11	0.5377	0.5377	0.5377	0.4107	0.2361	0.5694	0.2361	0.2679	0.0456	0.2520	0.0000	0.0298
A12	0.5694	0.2044	0.2361	0.4107	0.2520	0.2202	0.1409	0.5060	0.5694	0.3472	0.2361	0.0000

Normalize the direct relation matrix to get a normalized direct relation matrix. Then, Equation (10) is used to obtain the total relation matrix as shown in Table 4.

**TABLE 4:** The total relation matrix of the aspects.

	A1	A2	A3	A4	A5	A6	A7	A8	A9	A10	A11	A12
A1	0.1316	0.1871	0.1718	0.2681	0.1631	0.1623	0.2001	0.1701	0.1996	0.1967	0.1695	0.1725
A2	0.1888	0.1131	0.2241	0.2644	0.1174	0.1720	0.1954	0.1567	0.1768	0.1688	0.1870	0.2008
A3	0.1698	0.1671	0.1338	0.1732	0.1579	0.1428	0.1972	0.2564	0.2538	0.1344	0.1481	0.1740
A4	0.1156	0.0687	0.1102	0.0972	0.1483	0.0767	0.1049	0.1462	0.2199	0.1215	0.0807	0.1361
A5	0.2015	0.1744	0.2495	0.2693	0.1516	0.2263	0.2719	0.3001	0.2766	0.2198	0.2303	0.2882
A6	0.1531	0.1453	0.1750	0.1617	0.1389	0.1024	0.1444	0.2201	0.2507	0.1266	0.1553	0.1633
A7	0.3019	0.2393	0.3119	0.2720	0.2345	0.2473	0.1883	0.2873	0.2768	0.2359	0.2919	0.3037
A8	0.0869	0.1219	0.1546	0.1144	0.1215	0.1121	0.1285	0.0999	0.1272	0.1222	0.1080	0.1153
A9	0.0854	0.0486	0.0732	0.1160	0.1029	0.0580	0.0636	0.1068	0.0692	0.0531	0.0746	0.0817
A10	0.2358	0.2071	0.2662	0.2722	0.2063	0.2838	0.2878	0.2566	0.2881	0.1584	0.2858	0.2870
A11	0.2589	0.2455	0.2743	0.2618	0.1797	0.2562	0.2036	0.2275	0.1953	0.1873	0.1441	0.1631
A12	0.2553	0.1646	0.1981	0.2524	0.1809	0.1749	0.1713	0.2645	0.2891	0.1990	0.1808	0.1452

**FIGURE 1:**
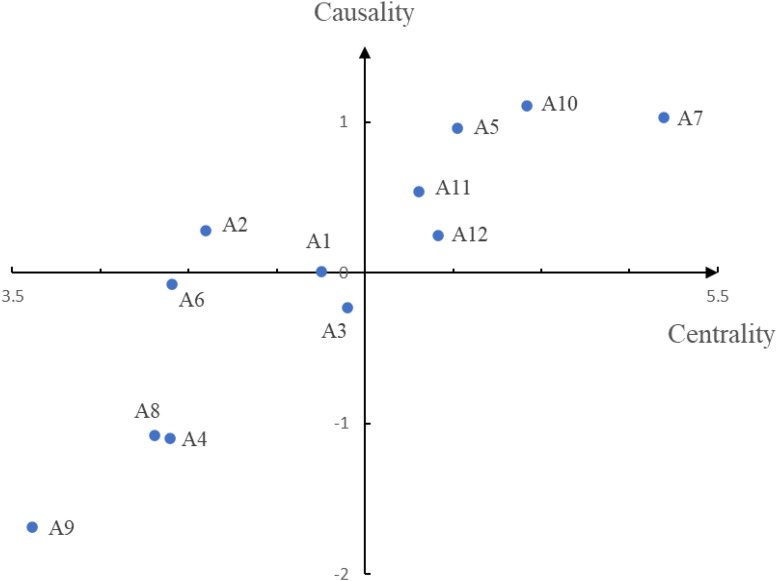
The cause and effect diagram of the aspects.

According to Equations (11)–(14), the driving power (D), dependence power (R), centrality (D+R), and causality (D−R) are calculated as shown in Table 5. The cause and effect diagram of the aspects is made as shown in Figure 1.

**TABLE 5:** Total relation matrix analysis of the aspects.

Aspects	D	R	(D+R)	(D−R)
A1	2.1924	2.1845	4.3769	0.0079
A2	2.1655	1.8828	4.0483	0.2826
A3	2.1084	2.3427	4.4512	−0.2343
A4	1.4261	2.5226	3.9487	−1.0966
A5	2.8595	1.9030	4.7625	0.9566
A6	1.9368	2.0150	3.9518	−0.0781
A7	3.1907	2.1570	5.3477	1.0337
A8	1.4126	2.4922	3.9048	−1.0796
A9	0.9330	2.6231	3.5561	−1.6901
A10	3.0352	1.9238	4.9589	1.1114
A11	2.5973	2.0561	4.6534	0.5412
A12	2.4762	2.2310	4.7072	0.2452

The driving power reflects the influence degree of the aspect on other aspects, and the dependence power indicates the influence degree of the aspect by other aspects. The causality clarifies the causal relationship between the aspects. The aspect with a positive causality has a greater influence on other aspects, and the aspect with a negative causality means that it is more affected by other aspects. The centrality reflects the position and importance of each aspect in the system. The greater the value, the more important the aspect is. The aspects with large values of both causality and centrality are located in the first quadrant of the cause-and-effect diagram (Figure 1). They are the aspects that can drive the achievement of other aspects and have a high degree of importance and need to be given priority in the case of limited resources.

According to the size of causality (D−R), 12 aspects are divided into the cause group and the effect group. As can be seen from Figure 1 and Table 5, seven aspects belong to the cause group, including production management (A1), logistics management (A2), psychology (A5), business opportunities (A7), institution (A10), financial management (A11), and leadership (A12). Among them, A5, A7, and A10 are the main driving aspects and have a great impact on other aspects. This is because entrepreneurship is inseparable from opportunities and institutions. The development of opportunities and the recognition from the system are the prerequisites for entrepreneurship. In addition, a strong psychological quality is the key to entrepreneurial success, and instilling entrepreneurial traits in the educated should be valued. In addition, there are five aspects in the effect group, including marketing management (A3), human resource management (A4), monetary decisions (A6), business ethics (A8), and corporate culture (A9). These aspects have a weak impact on entrepreneurship education and are more susceptible to other aspects. Therefore, these aspects should be given proper attention and control to help to improve the effect of entrepreneurship education. In terms of centrality, the priority of aspects is in the order of A7, A10, A5, A12, A11, A3, A1, A2, A6, A4, A8, and A9. Based on the above, it can be seen that A5, A7, and A10 are the crucial aspects, and the relevant measures should be taken into account.

Again, the CFCS method is used to process the original data of 29 criteria, and the direct relation matrix of the criteria is generated as shown in Table 6.

**TABLE 6:** The direct relation matrix of the criteria.

	C1	C2	C3	C4	C5	C6	C7	C8	C9	C10	C11	C12	C13	C14	C15	C16	C17	C18	C19	C20	C21	C22	C23	C24	C25	C26	C27	C28	C29
C1	0.00	0.01	0.01	0.08	0.11	0.09	0.10	0.06	0.06	0.07	0.05	0.04	0.08	0.08	0.07	0.14	0.22	0.22	0.01	0.01	0.05	0.04	0.12	0.05	0.15	0.22	0.24	0.22	0.24
C2	0.03	0.00	0.05	0.10	0.14	0.09	0.16	0.06	0.07	0.14	0.08	0.05	0.16	0.15	0.06	0.20	0.22	0.24	0.03	0.03	0.09	0.08	0.16	0.09	0.21	0.22	0.24	0.24	0.24
C3	0.01	0.01	0.00	0.10	0.10	0.06	0.09	0.04	0.05	0.07	0.05	0.03	0.08	0.08	0.06	0.14	0.22	0.22	0.01	0.01	0.04	0.04	0.11	0.05	0.15	0.22	0.22	0.20	0.20
C4	0.18	0.13	0.21	0.00	0.04	0.12	0.16	0.01	0.01	0.05	0.07	0.03	0.10	0.12	0.09	0.10	0.20	0.20	0.11	0.10	0.03	0.01	0.15	0.08	0.20	0.24	0.24	0.09	0.10
C5	0.16	0.10	0.16	0.01	0.00	0.10	0.14	0.01	0.01	0.02	0.06	0.04	0.06	0.09	0.05	0.08	0.16	0.16	0.08	0.08	0.01	0.01	0.12	0.06	0.16	0.24	0.24	0.06	0.06
C6	0.07	0.04	0.06	0.19	0.20	0.00	0.04	0.04	0.05	0.11	0.13	0.14	0.21	0.16	0.10	0.21	0.22	0.24	0.13	0.12	0.05	0.02	0.21	0.13	0.18	0.22	0.22	0.12	0.11
C7	0.05	0.03	0.05	0.12	0.19	0.01	0.00	0.01	0.03	0.07	0.12	0.10	0.14	0.10	0.06	0.14	0.22	0.22	0.12	0.09	0.02	0.01	0.17	0.09	0.15	0.19	0.22	0.08	0.08
C8	0.24	0.17	0.24	0.21	0.24	0.06	0.10	0.00	0.01	0.05	0.16	0.15	0.20	0.10	0.04	0.08	0.12	0.13	0.07	0.04	0.11	0.09	0.17	0.13	0.16	0.18	0.18	0.16	0.18
C9	0.24	0.24	0.24	0.24	0.24	0.06	0.11	0.01	0.00	0.05	0.16	0.16	0.21	0.10	0.05	0.07	0.13	0.13	0.04	0.07	0.12	0.11	0.18	0.12	0.15	0.20	0.21	0.17	0.17
C10	0.24	0.15	0.24	0.17	0.22	0.03	0.05	0.01	0.01	0.00	0.11	0.10	0.16	0.06	0.05	0.05	0.10	0.10	0.04	0.03	0.08	0.08	0.14	0.08	0.14	0.18	0.18	0.16	0.15
C11	0.10	0.08	0.10	0.01	0.01	0.09	0.11	0.14	0.14	0.18	0.00	0.01	0.03	0.10	0.05	0.11	0.17	0.17	0.10	0.10	0.10	0.10	0.12	0.06	0.06	0.11	0.10	0.08	0.10
C12	0.13	0.11	0.13	0.02	0.04	0.10	0.14	0.13	0.14	0.24	0.01	0.00	0.05	0.12	0.06	0.12	0.24	0.22	0.13	0.12	0.15	0.15	0.14	0.10	0.11	0.14	0.12	0.14	0.14
C13	0.08	0.08	0.08	0.01	0.01	0.05	0.10	0.10	0.12	0.14	0.01	0.01	0.00	0.05	0.05	0.09	0.14	0.15	0.06	0.07	0.06	0.06	0.13	0.05	0.07	0.08	0.08	0.07	0.08
C14	0.08	0.05	0.08	0.08	0.12	0.05	0.06	0.01	0.01	0.03	0.14	0.12	0.16	0.00	0.01	0.09	0.15	0.14	0.06	0.05	0.01	0.01	0.24	0.16	0.17	0.22	0.22	0.07	0.07
C15	0.11	0.06	0.10	0.10	0.14	0.06	0.10	0.01	0.01	0.05	0.13	0.15	0.24	0.05	0.00	0.13	0.20	0.22	0.06	0.06	0.05	0.02	0.24	0.24	0.22	0.24	0.24	0.13	0.10
C16	0.21	0.16	0.21	0.15	0.16	0.16	0.18	0.04	0.04	0.12	0.22	0.21	0.24	0.11	0.05	0.00	0.03	0.05	0.13	0.11	0.05	0.03	0.24	0.24	0.10	0.12	0.13	0.09	0.08
C17	0.14	0.10	0.13	0.09	0.15	0.12	0.16	0.04	0.04	0.05	0.20	0.16	0.24	0.06	0.04	0.01	0.00	0.01	0.06	0.07	0.01	0.01	0.24	0.16	0.05	0.10	0.09	0.05	0.06
C18	0.15	0.12	0.14	0.10	0.14	0.11	0.16	0.04	0.04	0.05	0.21	0.17	0.24	0.06	0.05	0.01	0.01	0.00	0.08	0.06	0.01	0.01	0.24	0.16	0.06	0.10	0.10	0.05	0.06
C19	0.06	0.05	0.05	0.02	0.03	0.22	0.24	0.07	0.08	0.15	0.20	0.16	0.24	0.20	0.17	0.10	0.18	0.18	0.00	0.01	0.10	0.08	0.12	0.07	0.06	0.12	0.12	0.21	0.20
C20	0.05	0.06	0.05	0.02	0.03	0.17	0.24	0.08	0.08	0.13	0.18	0.17	0.24	0.18	0.13	0.08	0.18	0.20	0.01	0.00	0.08	0.10	0.12	0.06	0.06	0.12	0.14	0.18	0.21
C21	0.10	0.06	0.08	0.03	0.06	0.22	0.24	0.21	0.22	0.24	0.14	0.10	0.24	0.24	0.20	0.07	0.14	0.14	0.06	0.05	0.00	0.01	0.20	0.12	0.01	0.03	0.05	0.22	0.24
C22	0.09	0.04	0.08	0.02	0.04	0.15	0.22	0.18	0.22	0.24	0.15	0.10	0.24	0.24	0.16	0.05	0.12	0.12	0.05	0.05	0.01	0.00	0.20	0.10	0.01	0.03	0.03	0.21	0.21
C23	0.14	0.10	0.12	0.08	0.10	0.05	0.05	0.08	0.08	0.09	0.13	0.12	0.17	0.06	0.05	0.10	0.16	0.17	0.08	0.07	0.01	0.01	0.00	0.01	0.06	0.10	0.10	0.10	0.11
C24	0.21	0.16	0.20	0.11	0.15	0.05	0.09	0.09	0.09	0.14	0.14	0.12	0.24	0.09	0.03	0.12	0.18	0.21	0.12	0.12	0.03	0.01	0.02	0.00	0.10	0.12	0.15	0.20	0.20
C25	0.12	0.06	0.12	0.14	0.18	0.08	0.12	0.01	0.01	0.04	0.14	0.14	0.21	0.16	0.10	0.22	0.24	0.24	0.09	0.08	0.09	0.05	0.24	0.24	0.00	0.04	0.03	0.13	0.12
C26	0.07	0.05	0.07	0.11	0.14	0.05	0.08	0.01	0.01	0.01	0.14	0.12	0.17	0.12	0.06	0.14	0.24	0.24	0.05	0.05	0.05	0.05	0.24	0.16	0.01	0.00	0.01	0.08	0.09
C27	0.08	0.05	0.07	0.12	0.15	0.05	0.09	0.01	0.01	0.01	0.14	0.10	0.17	0.11	0.05	0.14	0.24	0.24	0.03	0.04	0.05	0.04	0.24	0.16	0.01	0.01	0.00	0.09	0.10
C28	0.06	0.04	0.07	0.11	0.18	0.07	0.12	0.07	0.10	0.11	0.24	0.14	0.24	0.14	0.06	0.14	0.24	0.24	0.05	0.05	0.05	0.05	0.22	0.16	0.01	0.03	0.02	0.00	0.01
C29	0.07	0.05	0.07	0.12	0.20	0.07	0.12	0.08	0.09	0.12	0.24	0.16	0.24	0.15	0.08	0.16	0.24	0.24	0.05	0.05	0.05	0.05	0.22	0.16	0.01	0.02	0.04	0.01	0.00

Next, Equations (9) and (10) are used to get the total relation matrix of the criteria as shown in Table 7.

**TABLE 7:** The total relation matrix of the criteria.

	C1	C2	C3	C4	C5	C6	C7	C8	C9	C10	C11	C12	C13	C14	C15	C16	C17	C18	C19	C20	C21	C22	C23	C24	C25	C26	C27	C28	C29
C1	0.03	0.02	0.03	0.04	0.06	0.04	0.05	0.03	0.03	0.04	0.05	0.04	0.06	0.05	0.03	0.06	0.09	0.09	0.02	0.02	0.02	0.02	0.07	0.04	0.05	0.08	0.08	0.07	0.08
C2	0.05	0.03	0.05	0.05	0.07	0.05	0.07	0.03	0.03	0.06	0.06	0.05	0.09	0.07	0.03	0.08	0.10	0.11	0.03	0.03	0.04	0.03	0.09	0.06	0.07	0.09	0.09	0.08	0.09
C3	0.03	0.02	0.03	0.04	0.05	0.03	0.05	0.02	0.02	0.04	0.05	0.03	0.06	0.04	0.03	0.05	0.09	0.09	0.02	0.02	0.02	0.02	0.07	0.04	0.05	0.07	0.07	0.07	0.07
C4	0.07	0.05	0.07	0.03	0.05	0.05	0.07	0.02	0.02	0.03	0.05	0.04	0.07	0.06	0.04	0.05	0.09	0.09	0.04	0.04	0.02	0.01	0.08	0.05	0.07	0.08	0.09	0.05	0.05
C5	0.06	0.04	0.06	0.02	0.03	0.04	0.06	0.01	0.01	0.02	0.04	0.03	0.05	0.04	0.03	0.04	0.07	0.07	0.03	0.03	0.01	0.01	0.07	0.04	0.05	0.08	0.08	0.04	0.04
C6	0.06	0.04	0.05	0.07	0.08	0.03	0.05	0.03	0.03	0.05	0.07	0.07	0.10	0.07	0.04	0.08	0.10	0.11	0.05	0.05	0.03	0.02	0.10	0.07	0.07	0.09	0.09	0.06	0.06
C7	0.04	0.03	0.04	0.05	0.07	0.02	0.03	0.02	0.02	0.04	0.06	0.05	0.07	0.05	0.03	0.06	0.09	0.09	0.04	0.04	0.02	0.01	0.08	0.05	0.05	0.07	0.08	0.05	0.05
C8	0.09	0.06	0.09	0.07	0.09	0.04	0.06	0.02	0.02	0.04	0.08	0.07	0.10	0.06	0.03	0.06	0.09	0.09	0.04	0.03	0.04	0.03	0.09	0.07	0.06	0.08	0.08	0.07	0.08
C9	0.09	0.08	0.09	0.08	0.09	0.04	0.07	0.02	0.02	0.05	0.08	0.07	0.10	0.06	0.03	0.06	0.09	0.09	0.03	0.04	0.04	0.04	0.10	0.07	0.07	0.09	0.09	0.08	0.08
C10	0.08	0.05	0.08	0.06	0.08	0.03	0.05	0.02	0.02	0.03	0.06	0.05	0.08	0.04	0.03	0.04	0.07	0.07	0.03	0.03	0.03	0.03	0.08	0.05	0.05	0.07	0.08	0.07	0.07
C11	0.05	0.04	0.05	0.03	0.04	0.04	0.05	0.04	0.04	0.06	0.04	0.03	0.05	0.05	0.03	0.05	0.08	0.08	0.04	0.04	0.03	0.03	0.07	0.04	0.04	0.06	0.06	0.05	0.05
C12	0.06	0.05	0.06	0.04	0.05	0.05	0.07	0.04	0.05	0.08	0.05	0.04	0.07	0.06	0.04	0.06	0.10	0.10	0.05	0.05	0.05	0.04	0.09	0.06	0.05	0.07	0.07	0.07	0.07
C13	0.04	0.03	0.04	0.02	0.03	0.03	0.05	0.03	0.04	0.05	0.03	0.02	0.04	0.03	0.02	0.04	0.06	0.06	0.03	0.03	0.02	0.02	0.06	0.03	0.03	0.04	0.04	0.04	0.04
C14	0.04	0.03	0.04	0.04	0.05	0.03	0.04	0.01	0.02	0.03	0.06	0.05	0.07	0.03	0.02	0.05	0.07	0.07	0.03	0.03	0.02	0.01	0.09	0.06	0.06	0.07	0.08	0.04	0.04
C15	0.06	0.04	0.06	0.05	0.07	0.04	0.06	0.02	0.02	0.04	0.07	0.06	0.10	0.04	0.02	0.06	0.09	0.10	0.04	0.03	0.03	0.02	0.10	0.08	0.07	0.09	0.09	0.06	0.06
C16	0.08	0.06	0.08	0.06	0.07	0.06	0.08	0.03	0.03	0.06	0.09	0.08	0.10	0.06	0.03	0.04	0.07	0.07	0.05	0.05	0.03	0.02	0.10	0.08	0.06	0.07	0.07	0.06	0.06
C17	0.06	0.04	0.05	0.04	0.06	0.04	0.06	0.02	0.02	0.03	0.07	0.06	0.09	0.04	0.02	0.03	0.05	0.05	0.03	0.03	0.02	0.01	0.09	0.06	0.03	0.05	0.05	0.04	0.04
C18	0.06	0.04	0.06	0.04	0.06	0.04	0.06	0.02	0.02	0.03	0.07	0.06	0.09	0.04	0.03	0.03	0.05	0.05	0.03	0.03	0.02	0.01	0.09	0.06	0.04	0.05	0.06	0.04	0.04
C19	0.05	0.04	0.05	0.04	0.05	0.07	0.09	0.03	0.04	0.06	0.08	0.07	0.10	0.08	0.05	0.06	0.09	0.09	0.03	0.02	0.04	0.03	0.08	0.05	0.04	0.07	0.07	0.08	0.08
C20	0.05	0.04	0.05	0.03	0.05	0.06	0.08	0.03	0.04	0.06	0.08	0.07	0.10	0.07	0.05	0.05	0.09	0.09	0.02	0.02	0.03	0.03	0.08	0.05	0.04	0.07	0.07	0.07	0.08
C21	0.06	0.04	0.06	0.04	0.06	0.07	0.09	0.06	0.07	0.08	0.07	0.06	0.11	0.08	0.06	0.05	0.09	0.09	0.04	0.03	0.02	0.02	0.10	0.06	0.04	0.05	0.06	0.08	0.09
C22	0.06	0.04	0.05	0.04	0.05	0.05	0.08	0.05	0.06	0.08	0.07	0.05	0.10	0.08	0.05	0.04	0.08	0.08	0.03	0.03	0.02	0.02	0.09	0.06	0.03	0.05	0.05	0.08	0.08
C23	0.06	0.04	0.05	0.04	0.05	0.03	0.04	0.03	0.03	0.04	0.06	0.05	0.07	0.04	0.02	0.04	0.07	0.07	0.03	0.03	0.02	0.01	0.04	0.03	0.04	0.05	0.05	0.05	0.05
C24	0.08	0.06	0.08	0.05	0.07	0.04	0.06	0.04	0.04	0.06	0.07	0.06	0.10	0.05	0.03	0.06	0.09	0.10	0.05	0.04	0.02	0.02	0.06	0.04	0.05	0.07	0.07	0.08	0.08
C25	0.06	0.04	0.06	0.06	0.08	0.04	0.06	0.02	0.02	0.04	0.07	0.06	0.10	0.07	0.04	0.08	0.10	0.10	0.04	0.04	0.03	0.02	0.10	0.08	0.03	0.05	0.05	0.06	0.06
C26	0.04	0.03	0.04	0.04	0.06	0.03	0.05	0.02	0.02	0.03	0.06	0.05	0.07	0.05	0.03	0.05	0.09	0.09	0.03	0.03	0.02	0.02	0.09	0.06	0.02	0.03	0.03	0.04	0.05
C27	0.04	0.03	0.04	0.04	0.06	0.03	0.05	0.02	0.02	0.03	0.06	0.05	0.07	0.05	0.03	0.05	0.09	0.09	0.02	0.02	0.02	0.02	0.09	0.06	0.02	0.03	0.03	0.05	0.05
C28	0.05	0.03	0.05	0.05	0.07	0.04	0.06	0.03	0.04	0.05	0.08	0.06	0.09	0.06	0.03	0.06	0.09	0.09	0.03	0.03	0.02	0.02	0.09	0.06	0.03	0.04	0.04	0.03	0.04
C29	0.05	0.04	0.05	0.05	0.07	0.04	0.06	0.03	0.04	0.05	0.08	0.06	0.10	0.06	0.03	0.06	0.09	0.10	0.03	0.03	0.03	0.02	0.09	0.07	0.03	0.04	0.05	0.04	0.04

Finally, Equations (11)–(14) are used to calculate the driving power (D), dependence power (R), centrality (D+R), and causality (D−R), and the results are shown in Table 8.

**TABLE 8:** Total relation matrix analysis of the criteria.

Criteria	D	R	(D+R)	(D−R)
C1	1.6322	1.3934	3.0256	0.2388
C2	1.1701	1.7798	2.9499	−0.6097
C3	1.5922	1.2952	2.8874	0.2970
C4	1.3073	1.5360	2.8434	−0.2287
C5	1.7666	1.2164	2.9831	0.5502
C6	1.2254	1.8165	3.0420	−0.5911
C7	1.7398	1.3949	3.1347	0.3448
C8	0.7946	1.8417	2.6363	−1.0471
C9	0.8860	1.9336	2.8197	−1.0476
C10	1.3364	1.5101	2.8465	−0.1738
C11	1.8660	1.3320	3.1980	0.5340
C12	1.5268	1.7263	3.2530	−0.1995
C13	2.4180	1.0683	3.4863	1.3497
C14	1.5727	1.2808	2.8536	0.2919
C15	0.9440	1.6690	2.6130	−0.7249
C16	1.5388	1.8006	3.3394	−0.2618
C17	2.4235	1.2782	3.7017	1.1453
C18	2.4844	1.3225	3.8069	1.1619
C19	0.9793	1.7217	2.7010	−0.7424
C20	0.9245	1.6590	2.5835	−0.7345
C21	0.7515	1.8315	2.5830	−1.0801
C22	0.6304	1.6577	2.2882	−1.0273
C23	2.4476	1.2345	3.6821	1.2130
C24	1.6370	1.7079	3.3449	−0.0710
C25	1.3746	1.6772	3.0518	−0.3027
C26	1.8752	1.2631	3.1383	0.6120
C27	1.9272	1.2549	3.1822	0.6723
C28	1.6980	1.4775	3.1755	0.2205
C29	1.7449	1.5343	3.2792	0.2106

**FIGURE 2:**
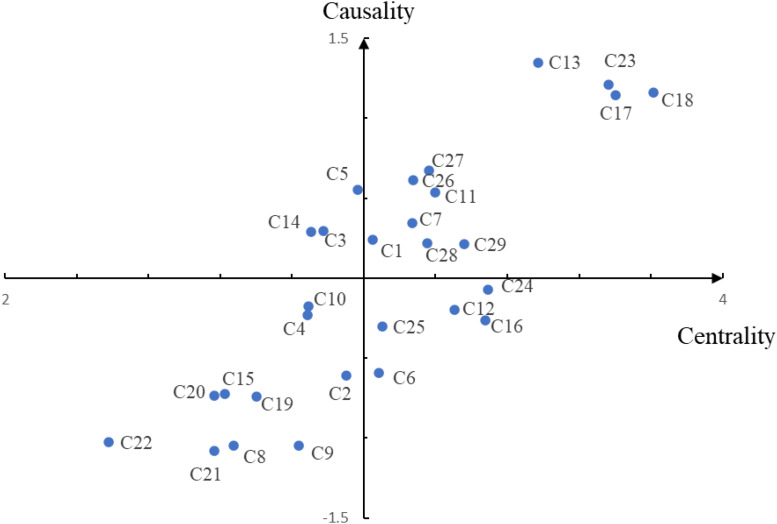
The cause and effect diagram of the criteria.

The cause-and-effect diagram of the criteria is made as shown in Figure 2.

As can be seen from Figure 2 and Table 8, there are 14 criteria that belong to the cause group, including C1, C3, C5, C7, C11, C13, C14, C17, C18, C23, C26, C27, C28, and C29, and C2, C4, C6, C8, C9, C10, C12, C15, C16, C19, C20, C21, C22, C24, and C25 are in the effect group. In addition, the cultivation of self-efficacy (C13) under A5, the identification of discovery opportunities (C17) and creative opportunities (C18) under A7, and formal institutional entrepreneurship (C23) under A10 are four important driving criteria of entrepreneurship education with the application of big data technology.

After that, the ISM method is used to get the hierarchical framework. Based on Equations (15)–(18) and the total relation matrix of the aspects, the reachability matrix is generated as shown in Table 9.

**TABLE 9:** The reachability matrix.

	A1	A2	A3	A4	A5	A6	A7	A8	A9	A10	A11	A12
A1	1	0	0	1	0	0	0	0	0	0	0	0
A2	0	1	0	1	0	0	0	0	0	0	0	0
A3	0	0	1	0	0	0	0	1	1	0	0	0
A4	0	0	0	1	0	0	0	0	0	0	0	0
A5	0	0	1	1	1	0	1	1	1	0	0	1
A6	0	0	0	0	0	1	0	0	1	0	0	0
A7	1	0	1	1	0	0	1	1	1	0	1	1
A8	0	0	0	0	0	0	0	1	0	0	0	0
A9	0	0	0	0	0	0	0	0	1	0	0	0
A10	0	0	1	1	0	1	1	1	1	1	1	1
A11	1	0	1	1	0	1	0	0	0	0	1	0
A12	1	0	0	1	0	0	0	1	1	0	0	1

Then the reachability set L(fi), antecedent set P(fi), and intersection set C(fi) are obtained from the reachability matrix and Equation (19) as shown in Table 10.

**TABLE 10:** Primary decomposition structure.

*i* (Aspects)	*L(f_*i*_)*	*P*(*f*_*i*_)	*C(f_*i*_)* = *L(f_*i*_)*∩*P(f_*i*_*)
(1) (A1)	1,4	1,7,11,12	1
(2) (A2)	2,4	2	2
(3) (A3)	3,8,9	3,5,7,10,11	3
(4) (A4)	4	1,2,4,5,7,10,11,12	4
(5) (A5)	3,4,5,7,8,9,12	5	5
(6) (A6)	6,9	6,10,11	6
(7) (A7)	1,3,4,7,8,9,11,12	5,7,10	7
(8) (A8)	8	3,5,7,8,10,12	8
(9) (A9)	9	3,5,6,7,9,10,12	9
(10) (A10)	3,4,6,7,8,9,10,11,12	10	10
(11) (A11)	1,3,4,6,11	7,10,11	11
(12) (A12)	1,4,8,9,12	5,7,10,12	12

**FIGURE 3:**
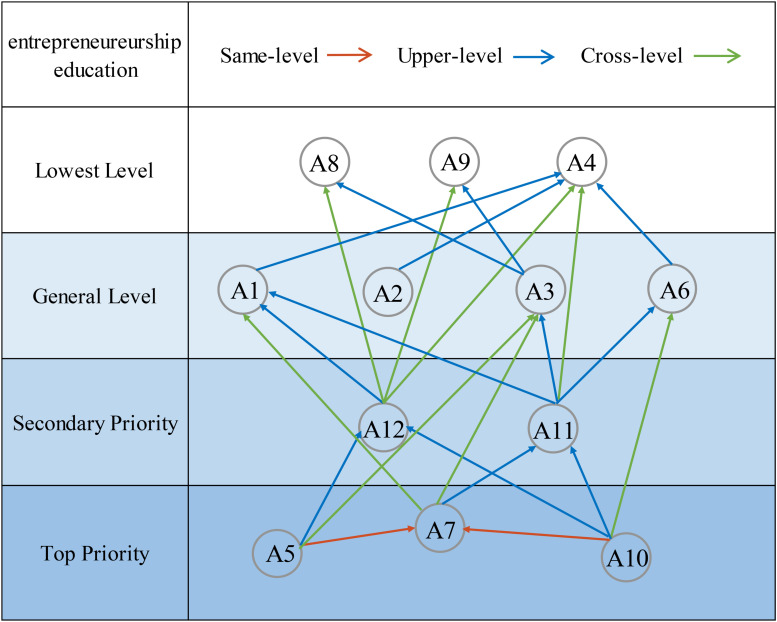
ISM model.

As can be seen from Table 10, the reachability set and the antecedent set intersect in three aspects of A4, A8, and A9, which constitute a level of the hierarchical framework. The rows and columns mapped by A4, A8, and A9 in the reachability matrix are deleted to obtain a higher-level decomposition structure, and the above processes are performed repeatedly. After several hierarchical divisions, the factor set Nq (*q* = 1, 2,…, 5) of each layer is finally obtained: N1 = {A4, A8, A9}; N2 = {A1, A2, A3, A6}; N3 = {A11, A12}; N4 = {A7}; N5 = {A5, A10}. Based on the above analysis, the ISM model is established and shown in Figure 3. It can be seen that A5, A7, and A10 are the rooted aspects of entrepreneurship education affecting other aspects and should be given priority consideration.

In summary, the attributes that affect entrepreneurship education are complex, and there are interactions between each level. Different attributes have different influencing modes and mechanisms, thus forming a systematic integration framework of entrepreneurship education under big data.

## Discussion and Conclusion

### Discussion

There are few studies on the application of big data technology to the field of entrepreneurship education. This study systematically proposes a set of criteria for the development of entrepreneurship education and offers a new hierarchical theoretical framework. The results reveal that the hierarchical theoretical framework can be divided into four levels.

Throughout the framework, business opportunities (A7), psychology (A5), and institution (A10) are at the first level. Although these are three different aspects of entrepreneurship education, they are not isolated from each other and reflect the characteristics of entrepreneurship indirectly. First, the identification of business opportunities is at the first level, which is in line with core entrepreneurship theory. Entrepreneurship itself is a process of chasing and realizing business opportunities (Shane and Venkataraman, 2000). As the external environmental factor of entrepreneurship, the aspect of the institution is at the first level, which is consistent with the theory of legitimacy (Zimmerman and Zeitz, 2002). The theory of legitimacy emphasizes that the success of institutional entrepreneurship depends on the acquisition of legitimacy, and new ventures must surmount the obstacle of legitimacy and be recognized by the system. There is a certain correlation between the aspects of institution and business opportunities in this paper. The results show that institutional education has an impact on the identification and acquisition of business opportunities. Finally, the aspect of psychology is at the first level, which is consistent with existing research on entrepreneurial psychology (Ismail and Zain, 2015). Psychological education is related to the personal characteristics of entrepreneurs, which influences the identification of opportunities and is the key to the success of entrepreneurship education. It can be seen that, at the first level, the results not only validate the theory of institutional entrepreneurship (Maguire et al., 2004; Greenwood and Suddaby, 2006), but also verify the importance of developing opportunities and instilling entrepreneurial traits in entrepreneurship education.

As a new aspect, leadership (A12) has been developed in this paper, which is relatively rare in the field of entrepreneurship education. This aspect has a deep relationship with behavioral theories of leadership, emphasizing that the leadership behavior of entrepreneurs has an important impact on corporate performance (Chandler and Hanks, 1994). The development of this aspect needs to use big data technology to study successful leadership cases and explore the best ways to achieve leadership effectiveness to enhance transactional leadership and transformational leadership. The two aspects of leadership and psychology are closely related, and they are, respectively, associated with the behaviors and traits of leaders. The results show that they are at a relatively high level, which is consistent with the upper echelons theory. The upper echelons theory claims that the organization is the reflection of its top managers, whose values, characteristics, and behaviors play a decisive role in the strategic choice and organizational performance of the enterprise (Hambrick and Mason, 1984; Tecle and Ayako, 2016; Georgakakis et al., 2017). The results of this paper not only reflect the combination of behavioral theories and trait theory of leadership, but also prove the importance of upper managers’ traits and entrepreneurs’ leadership behaviors in organizations (Marshall et al., 1995). Apart from the aspect of leadership discussed above, financial management (A11) is also located on the second level and is identified as a significant aspect. It precedes processes of value creating in entrepreneurship and is valued by entrepreneurship education. Financial management stresses the integration of the financial department with other departments, the forecast of financial conditions, and the assessment and avoidance of financial risks, which reflects the importance that entrepreneurs place on cash flow and the need for risk defense (Forlani and Mullins, 2000).

The third level is the development stage of formal entrepreneurial activities, which is a process of developing opportunities and creating value. Hence, this level is mainly based on the theory of the value chain, involving the four aspects of production management (A1), logistics management (A2), marketing management (A3), and monetary decisions (A6). All links complement each other and have vital connections. For example, feedback from customers reveals new demand, which is part of the identification of business opportunities and can provide corresponding information for the adjustment of corporate production plans so that the products can meet customer demand. In addition, in the operation process of value creating, the role of big data technology is mainly related to reducing costs, ensuring quality, interacting and sharing information, and optimizing decisions, which maximizes the value created by the enterprise. In addition, the results reveal that the activities of value creating are affected by aspects located on the first two levels. Entrepreneurship theory emphasizes that the success of entrepreneurial activities depends on entrepreneurs (McGrath and MacMillan, 2000), and the realization of value creating is influenced by the behavior of leaders. Apart from this, there is an important correlation between value-creating activities of enterprises and the external institutional environment, which may explain why uncertain institutional factors are significant factors in economic development. Finally, the results indicate that the value-creating processes are affected by the types of business opportunities and the identification and evaluation of business opportunities. The realization of corporate value-creating activities depends on the identification of different types of opportunities and the acquisition and integration of resources (Sirmon et al., 2007).

Human resource management (A4), business ethics (A8), and corporate culture (A9) are at the last level. The construction of these three aspects requires the participation and cooperation of all employees, which needs to go through a long and complex process and is affected by aspects on the first three levels. Human resource management is at this level, reflecting the long-term nature of human resource development. Additionally, the construction of business ethics and corporate culture is mostly related to awareness of corporate social responsibility and environmental protection, which is an important way for companies to gain social benefits. Business ethics and corporate culture are at the last level, which also shows that the shaping of ethics and values is still behind economic construction. It is related to the nature of entrepreneurship. Entrepreneurship itself is different from operating large mature enterprises. For start-ups, the main goals of the early stage are still to create economic benefits to maintain the survival and development of the enterprise (Gao et al., 2018). However, if we expand our vision to mature enterprises, social and economic benefits may be equally important, which reflects the pursuit of long-term interests and is in line with the theory of corporate social responsibility.

### Implications

The research of this paper has some implications. First of all, this paper has certain theoretical implications. The study considers the theories of entrepreneurship (development of business opportunities, acquisition of legitimacy, etc.), leadership (traits and behaviors), and strategic management (value chain model). By sorting out the existing literature, the attribute system is constructed, covering 12 aspects and 29 criteria related to entrepreneurship education, which is innovative and forward-looking. Second, in terms of methodological implications, the fuzzy-DEMATEL hybrid method is used to evaluate the complex relationship among 12 aspects and 29 criteria, and the key driving aspects and criteria in entrepreneurship education are revealed, which helps to manage the multiattribute decision making of entrepreneurship education. At the same time, the use of fuzzy set theory overcomes the problem of linguistic preferences, and the credibility of the results is enhanced. In addition, the research divides 12 aspects of entrepreneurship education into four levels by using the ISM method, clarifies the influence paths among the levels, and finally constructs a systematic and clear hierarchical framework, which is helpful for managers to conduct overall management and control of entrepreneurship education from a macro perspective. Finally, the results show that the attributes of entrepreneurship education under big data technology can be divided into four levels, which can also clearly and systematically reveal the priority of the different levels. The results of this paper are not only the verification of classic theories, but also the innovative guidance of entrepreneurship education, which is of great significance to the sustainable development of entrepreneurs and new ventures.

### Limitations and Future Research

Previous research on entrepreneurship education focuses on entrepreneurship education in college at the traditional level, lacking quantitative research and a systematic framework. Compared with previous studies, this paper considers the upgrading and challenges to entrepreneurship education brought by big data technology. In addition, the object of entrepreneurship education in this article becomes more extensive, and the research plays a guiding role for all entrepreneurs. This research is based on the integration of multiple theories and takes the embeddedness of new technologies into account, which can promote the integration and systematic development of entrepreneurship education theories and provide viable suggestions for the practical application of entrepreneurship education.

However, this study still has several limitations. First, although a systematic framework has been constructed in this article, there is still a possibility of missing attributes, which needs to be further explored in future research. Second, the research is based on the questionnaire data from experts. Although fuzzy set theory is used to address linguistic preferences of experts, there are still some errors that are difficult to eliminate, which may have a certain impact on the results. Finally, the results of this paper have yet to be verified and supplemented in practice. Future research should focus on solving the above problems and constantly improve the thinking of entrepreneurship education.

## Data Availability Statement

The raw data supporting the conclusions of this article will be made available by the authors, without undue reservation.

## Ethics Statement

Ethical review and approval was not required for the study on human participants in accordance with the local legislation and institutional requirements. Written informed consent from the patients/participants was not required to participate in this study in accordance with the national legislation and the institutional requirements.

## Author Contributions

HM and CL provided the idea. YL and YG wrote the manuscript. HM and YL carried out the data analysis. All authors contributed to the article and approved the submitted version.

## Conflict of Interest

The authors declare that the research was conducted in the absence of any commercial or financial relationships that could be construed as a potential conflict of interest.

## References

[B1] Ahlstrom D. Bruton G. D. (2002). An institutional perspective on the role of culture in shaping strategic actions by technology-focused entrepreneurial firms in China. *Entrep. Theory Pract.* 26 53–68. 10.1177/104225870202600404

[B2] Anshari M. Almunawar M. N. Lim S. A. Al-Mudimigh A. (2019). Customer relationship management and big data enabled: personalization & customization of services. *Appl. Comput. Inform.* 15 94–101. 10.1016/j.aci.2018.05.004

[B3] Beikkhakhian Y. Javanmardi M. Karbasian M. Khayambashi B. (2015). The application of ISM model in evaluating agile suppliers selection criteria and ranking suppliers using fuzzy TOPSIS-AHP methods. *Expert Syst. Appl.* 42 6224–6236. 10.1016/j.eswa.2015.02.035

[B4] Büyüközkan G. Çifçi G. (2012). A novel hybrid MCDM approach based on fuzzy DEMATEL, fuzzy ANP and fuzzy TOPSIS to evaluate green suppliers. *Expert Syst. Appl.* 39 3000–3011. 10.1016/j.eswa.2011.08.162

[B5] Chandler G. N. Hanks S. H. (1994). Market attractiveness, resource-based capabilities, venture strategies, and venture performance. *J. Bus. Ventur.* 9 331–349. 10.1016/0883-9026(94)90011-6

[B6] Chen G. Liu Z. Sun Y. Liu W. Lv Q. Song Y. (2018). “Analysis of the impact of big data on enterprise decision making,” in *2018 8th International Conference on Social science and Education Research (SSER 2018)*, Amsterdam: Atlantis Press, 10.2991/sser-18.2018.45

[B7] Chen Y. Lou C. Cao M. Wu X. (2018). “The construction and exploration of the hierarchical innovation and entrepreneurship education system in colleges and universities,” in *International Conference on Contemporary Education, Social Sciences and Ecological Studies (CESSES 2018)*, Amsterdam: Atlantis Press, 10.2991/cesses-18.2018.62

[B8] Chen Q. (2017). “Exploration on recognition and construction of core competitiveness of enterprise culture,” in *2017 International Conference on Humanities Science, Management and Education Technology (HSMET 2017)*, Amsterdam: Atlantis Press, 10.2991/hsmet-17.2017.43

[B9] Cheng J. Y. Dong P. W. (2015). “Thinking of corporation financial management innovation in the era of big data,” in *2015 International Conference on Management Science and Management Innovation (MSMI 2015)*, Amsterdam: Atlantis Press, 10.2991/msmi-15.2015.83

[B10] Cui Y. Sun C. Xiao H. Zhao C. (2016). How to become an excellent entrepreneur: the moderating effect of risk propensity on alertness to business ideas and entrepreneurial capabilities. *Technol. Forecast. Soc. Chang.* 112 171–177. 10.1016/j.techfore.2016.08.002

[B11] Du X. Gao Y. Wu C. H. Wang R. Bi D. (2020). Blockchain-Based intelligent transportation: a sustainable GCU application system. *J. Adv. Transp.* 2020 1–14. 10.1155/2020/5036792

[B12] Findler F. Schönherr N. Lozano R. Reider D. Martinuzzi A. (2019). The impacts of higher education institutions on sustainable development: a review and conceptualization. *Int. J. Sustain. High. Educ.* 20 23–38. 10.1108/IJSHE-07-2017-0114

[B13] Forlani D. Mullins J. W. (2000). Perceived risks and choices in entrepreneurs’ new venture decisions. *J. Bus. Ventur.* 15 305–322. 10.1016/S0883-9026(98)00017-2

[B14] Gao Y. Ge B. Lang X. Xu X. (2018). Impacts of proactive orientation and entrepreneurial strategy on entrepreneurial performance: an empirical research. *Technol. Forecast. Soc. Chang.* 135 178–187. 10.1016/j.techfore.2017.11.019

[B15] García-Morales V. J. Jiménez-Barrionuevo M. M. Gutiérrez-Gutiérrez L. (2012). Transformational leadership influence on organizational performance through organizational learning and innovation. *J. Bus. Res.* 65 1040–1050. 10.1016/j.jbusres.2011.03.005

[B16] García-Rodríguez F. J. Gutiérrez-Taño D. Ruiz-Rosa I. (2017). The business model approach in entrepreneurship education: impact on undergraduates’ enterprise potential. *Mediterr. J. Soc. Sci.* 8 11–17. 10.5901/mjss.2017.v8n3p11

[B17] Georgakakis D. Greve P. Ruigrok W. (2017). Top management team faultlines and firm performance: examining the CEO-TMT interface. *Leadersh. Q.* 28 741–758. 10.1016/j.leaqua.2017.03.004

[B18] Ghasemaghaei M. Ebrahimi S. Hassanein K. (2018). Data analytics competency for improving firm decision-making performance. *J. Strateg. Inf. Syst.* 27 101–113. 10.1016/j.jsis.2017.10.001

[B19] Gianiodis P. T. Meek W. R. (2019). Entrepreneurial education for the entrepreneurial university: a stakeholder perspective. *J. Technol. Transf.* 45 1167–1195. 10.1007/s10961-019-09742-z

[B20] Greenwood R. Suddaby R. (2006). Institutional entrepreneurship in mature fields: the big five accounting firms. *Acad. Manage. J.* 49 27–48. 10.5465/amj.2006.20785498

[B21] Hambrick D. C. Mason P. A. (1984). Upper echelons: the organization as a reflection of its top managers. *Acad. Manage. Rev.* 9 193–206. 10.5465/amr.1984.4277628

[B22] Han Y. (2019). “Analysis of enterprise marketing innovation level and path based on big data perspective,” in *2019 3rd International Conference on Education, Management Science and Economics (ICEMSE 2019)*, Amsterdam: Atlantis Press, 10.2991/icemse-19.2019.130

[B23] Hao Y. (2017). “Research on building curriculum system of entrepreneurship education for college students in China,” in *2016 7th International Conference on Education, Management, Computer and Medicine (EMCM 2016)*, Amsterdam: Atlantis Press, 10.2991/emcm-16.2017.254

[B24] Hassan A. Gallear D. Sivarajah U. (2018). Critical factors affecting leadership: a higher education context. *Transf. Governm. People Process Policy* 12 110–130. 10.1108/TG-12-2017-0075

[B25] Hua Z. (2019). “Create an online and offline practice platform to establish a new model of innovation and entrepreneurship education,” in *2018 8th International Conference on Education and Management (ICEM 2018)*, Amsterdam: Atlantis Press, 10.2991/icem-18.2019.101

[B26] Ismail V. Y. Zain E. (2015). The portrait of entrepreneurial competence on student entrepreneurs. *Procedia Soc. Behav. Sci.* 169 178–188. 10.1016/j.sbspro.2015.01.300

[B27] Klašnja-Milićević A. Ivanović M. Budimac Z. (2017). Data science in education: big data and learning analytics. *Comput. Appl. Eng. Educ.* 25 1066–1078. 10.1002/cae.21844

[B28] Kubina M. Varmus M. Kubinova I. (2015). Use of big data for competitive advantage of company. *Proc. Econ. Financ.* 26 561–565. 10.1016/S2212-5671(15)00955-7

[B29] Li H. (2018). “Innovation of performance management of enterprise human resource in the era of big data,” in *2nd International Conference on Economics and Management, Education, Humanities and Social Sciences (EMEHSS 2018)*, Amsterdam: Atlantis Press, 10.2991/emehss-18.2018.117

[B30] Li L. Zhao L. (2019). “Design about cost management of logistics enterprises under the background of the big data and informatization,” in *2018 International Symposium on Social Science and Management Innovation (SSMI 2018)*, Amsterdam: Atlantis Press, 10.2991/ssmi-18.2019.72

[B31] Li J.-J. Li J. Zhu L. (2018). The disclosure of social responsibility information of coal enterprises in big data environment. *DEStech Trans. Econ. Bus. and Manag.* 4, 227–230. 10.12783/dtem/eced2018/23968

[B32] Li W. X. (2017). The research and construction of FCP college students’ entrepreneurship education system. *DEStech Trans. Soc. Sci. Educ. Hum. Sci.* 5, 133–138. 10.12783/dtssehs/emse2017/12756

[B33] Lin C.-L. Tzeng G.-H. (2009). A value-created system of science (technology) park by using DEMATEL. *Expert Syst. Appl.* 36 9683–9697. 10.1016/j.eswa.2008.11.040

[B34] Longshore J. M. Bass B. M. (1987). Leadership and performance beyond expectations. *Acad. Manage. Rev.* 12:756 10.2307/258081

[B35] Maguire S. Hardy C. Lawrence T. B. (2004). Institutional entrepreneurship in emerging fields: HIV/AIDS treatment advocacy in Canada. *Acad. Manage. J.* 47 657–679. 10.5465/20159610 20159610

[B36] Marshall J. N. Alderman N. Wong C. Thwaites A. (1995). The impact of management training and development on small and medium-sized enterprises. *Int. Small Bus J.* 13 73–90. 10.1177/0266242695134004

[B37] McGrath R. G. MacMillan I. C. (2000). Assessing technology projects using real options reasoning. *Res.-Technol. Manag.* 43 35–49. 10.1080/08956308.2000.11671367

[B38] Opricovic S. Tzeng G. H. (2004). Compromise solution by MCDM methods: a comparative analysis of VIKOR and TOPSIS. *Eur. J. Oper. Res.* 156 445–455. 10.1016/S0377-2217(03)00020-1

[B39] Rijmenam M. V. Erekhinskaya T. Schweitzer J. Williams M. A. (2019). Avoid being the Turkey: how big data analytics changes the game of strategy in times of ambiguity and uncertainty. *Long Range Plan.* 52:101841 10.1016/j.lrp.2018.05.007

[B40] Sarfraz M. Qun W. Shah S. G. M. Fareed Z. (2019). Do hierarchical Jumps in CEO succession invigorate innovation? Evidence from chinese economy. *Sustainability* 11:2017 10.3390/su11072017

[B41] Shah S. G. M. Sarfraz M. Fareed Z. ur Rehman M. A. Maqbool A. Qureshi M. A. A. (2019). Whether CEO succession via hierarchical jumps is detrimental or blessing in disguise? Evidence from Chinese listed firms. *Zagreb Int. Rev Econ. Bus.* 22 23–41. 10.2478/zireb-2019-0018

[B42] Shane S. Venkataraman S. (2000). The promise of entrepreneurship as a field of research. *Acad. Manage. Rev.* 25 217–226. 10.5465/amr.2000.2791611

[B43] Shen H. (2015). “Research on enterprise human resources management mode innovation in the age of big data,” in *2015 International Conference on Economics, Management, Law and Education*, Amsterdam: Atlantis Press, 10.2991/emle-15.2015.73

[B44] Shen Y. (2018). “On strengthening the construction of enterprise culture to improve enterprise competitiveness,” in *2018 5th International Conference on Education, Management, Arts, Economics and Social Science (ICEMAESS 2018)*, Amsterdam: Atlantis Press, 10.2991/icemaess-18.2018.171

[B45] Sirmon D. G. Hitt M. A. Ireland R. D. (2007). Managing firm resources in dynamic environments to create value: looking inside the black box. *Acad. Manage. Rev.* 32 273–292. 10.5465/amr.2007.23466005

[B46] Tecle H. Y. Ayako A. B. (2016). Top management team demographic diversities, generic strategy and firm performance in marketing social research association (msra) in kenya. *Appl. Financ. Account* 2 30–45.

[B47] Wang K. (2018). “The innovation of enterprise management mode under the background of big data,” in *3rd International Conference on Contemporary Education, Social Sciences and Humanities (ICCESSH 2018)*, Amsterdam: Atlantis Press, 10.2991/iccessh-18.2018.262

[B48] Wang Q. (2019). “Discussion on the challenges and innovative thinking of financial management under the background of big data,” in *3rd International Seminar on Education Innovation and Economic Management (SEIEM 2018)*, Amsterdam: Atlantis Press, 10.2991/seiem-18.2019.171

[B49] Wang Y. Hajli N. (2017). Exploring the path to big data analytics success in healthcare. *J. Bus. Res.* 70 287–299. 10.1016/j.jbusres.2016.08.002

[B50] Wu Y. J. Yuan C.-H. Pan C.-I. (2018). Entrepreneurship education: an experimental study with information and communication technology. *Sustainability* 10:691 10.3390/su10030691

[B51] Xia J. Zhou W. (2015). “A brief analysis of opportunities and challenges for accounting personnel in the big data era,” in *International Conference on Education, Management and Computing Technology (ICEMCT-15*, Amsterdam: Atlantis Press, 10.2991/icemct-15.2015.309

[B52] Yan G. (2019). “Discussion on the method of logistics cost control in foreign trade enterprises,” in *4th International Conference on Humanities Science, Management and Education Technology (HSMET 2019)*, Amsterdam: Atlantis Press, 10.2991/hsmet-19.2019.119

[B53] Yang B. Ge J. Song W. Y. (2014). “On the construction of the open-university innovation and entrepreneurship education system,” in *3rd International Conference on Science and Social Research (ICSSR 2014)*, Amsterdam: Atlantis Press, 10.2991/icssr-14.2014.117

[B54] Yu X. (2015). “Research on modelling novel Internet finance pattern under big data and risk-aware environment,” in *2015 International Conference on Education, Management, Information and Medicine*, Amsterdam: Atlantis Press, 10.2991/emim-15.2015.19

[B55] Zhang H. (2019). “Reflections on the innovation of human resources management in the era of big data,” in *2018 8th International Conference on Education and Management (ICEM 2018)*, Amsterdam: Atlantis Press, 10.2991/icem-18.2019.136

[B56] Zhang K. Xu P. (2018). “Research on transformation strategy of enterprise human resource management in big data era,” in *2018 International Conference on Management, Economics, Education and Social Sciences (MEESS 2018)*, Amsterdam: Atlantis Press, 10.2991/meess-18.2018.2

[B57] Zhang S. Xiong W. Ni W. Li X. (2015). Value of big data to finance: observations on an Internet credit service company in China. *Financial Innov.* 1 1–18. 10.1186/s40854-015-0017-2

[B58] Zheng D. (2019). “Construction of entrepreneurship education system for logistics management majors based on big data mining,” in *3rd International Seminar on Education Innovation and Economic Management (SEIEM 2018)*, Amsterdam: Atlantis Press, 10.2991/seiem-18.2019.42

[B59] Zhou Q. Gao S. (2019). “An empirical study on the relationship between entrepreneurial resources and entrepreneurial competence,” in *1st International Conference on Business, Economics, Management Science (BEMS 2019*, Amsterdam: Atlantis Press, 10.2991/bems-19.2019.71

[B60] Zhu W. Chew I. K. Spangler W. D. (2005). CEO transformational leadership and organizational outcomes: the mediating role of human–capital-enhancing human resource management. *The leadersh. Q.* 16 39–52. 10.1016/j.leaqua.2004.06.001

[B61] Zimmerman M. A. Zeitz G. J. (2002). Beyond survival: achieving new venture growth by building legitimacy. *Acad. Manage. Rev.* 27 414–431. 10.5465/amr.2002.7389921

